# Burning Characteristics and Smoke Emission from Mixed Fuel Cribs

**DOI:** 10.1021/acsestair.4c00275

**Published:** 2025-03-21

**Authors:** Aika Y. Davis, Thomas G. Cleary, Ryan L. Falkenstein-Smith, Rodney A. Bryant

**Affiliations:** † Fire Research Division, National Institute of Standards and Technology, Gaithersburg, Maryland 20899, United States

**Keywords:** structure fire, smoke, emission factors, mixed fuel crib, oxygen consumption calorimetry, FTIR

## Abstract

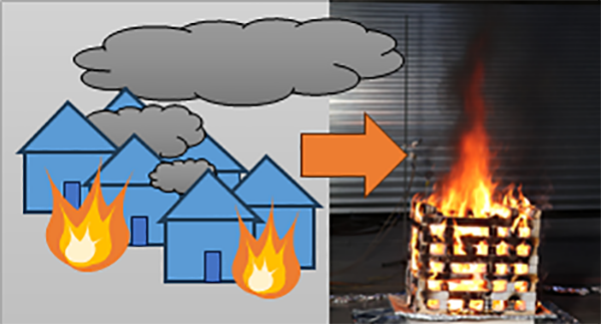

Experiments were conducted to study the burning characteristics and smoke emission from mixed fuel crib assemblies designed to represent the main components in structures. Cribs consisting of wood, drywall, and plastics were assembled into cubic structures, all with the same mass fraction for each material. Three packing densities and two crib sizes were studied. While a crib burned to completion, the smoke was collected in a fire calorimetry system for the heat release rate (HRR), combustion gases, and smoke measurements. The scale of cribs affected the burn duration and the total heat release, but the packing density had a greater effect on modified combustion efficiency, peak HRR, and the emission factors of CO_2_, CO, formaldehyde, acrolein, SO_2_, and total hydrocarbons. The variability in scale and packing density studied here can affect the structure fire emission estimates by 20% for CO_2_ and smoke and up to 130% for SO_2_ and formaldehyde. Therefore, packing density must be considered to properly evaluate structural fires.

## Abstract 2

Burning behavior and emission yields of mixed fuel fire found here could help quantify smoke production in wildland urban interface communities and aid in predicting smoke spread and deteriorating air quality downwind.

## Introduction

Wildland urban interface (WUI) fires have been increasing globally over the last few decades, presenting severe threats to public health,^1−5^ they can propagate and destroy communities and the smoke generated can deteriorate air quality and introduce health risks to adjacent regions for kilometers downwind.^1,3,6−8^ WUI fires can generate complex emissions different from wildland fires because they combine synthetic materials used for building structures, vehicles, and other infrastructure with natural biomass.^3^ Combustion and smoke characteristics of natural vegetation, wood products, and synthetic materials have been extensively studied separately, but little research has been conducted on mixed fuel combustion and quantifying WUI fire emission factors from the built environment.^4,5^

WUI structure fires can exhibit varying ventilation conditions with synthetic materials and wood products burning simultaneously with initial interior or exterior ignitions, extending through the structure until collapsing and/or completely consuming the fuels. These details vary from structure to structure and impact the burning rate and emission yields from structures, which presents a need to more fully characterize WUI fires.^6^

Bryner and Mulholland^9^ examined the effects of fuel mixtures and packing densities to assess smoke yields from building rubble. They measured heat release rate (HRR), mass loss rate, and smoke extinction for cribs, an ordered array of sticks, with varying combinations of wood, gypsum board, and acrylonitrile butadiene styrene (ABS).^9^ The crib configuration proved useful in examining fuel, configuration, and ventilation effects on smoke yield. Additionally, wood cribs have been used as models for wildland fires.^10^

As the first step toward experimentally characterizing WUI structural fire, mixed fuel cribs were constructed to represent simplified/idealized structures. Fire behavior, smoke characterization, and gas species yield of mixed fuel cribs were investigated. Cribs containing wood, oriented strand board (OSB), gypsum wallboard (Gyp), ABS, polyvinyl chloride (PVC), and polyurethane (PU) were constructed, stacking combustible and noncombustible materials evenly spread to mimic a structure fuel loading. The Gyp tends to maintain some structural integrity which retains the cubic shape of the crib until fuel components burn out. The cribs varied in size and packing density to study the effects of scale and ventilation. In the context of structure fires, low packing density cribs may be more representative of free-standing structures, while high packing density cribs are more representative of collapsed structures. HRR, modified combustion efficiency, average effective heat of combustion, and smoke and combustion product yields of cribs are presented.

This study was motivated by the need to obtain combustion characteristics and smoke yields of a burning structure and understand the effects of fuel scale and configuration (such as ventilation and packing density). Smoke particle mass (e.g., PM_2.5_), carbon monoxide (CO), hydrogen cyanide (HCN), nitrogen oxides (NO_*x*_), and formaldehyde (HCHO) are just a few smoke constituents known to pose health risks,^1^ and the yields (or emission factors) of these species are identified and monitored with respect to fuel configurations.

This study’s crib data can be compared to real-scale structure burn yields in the future and provide additional emissions inventory data for WUI fires. A repeatable structure fire smoke source is needed for ongoing indoor air quality and short and long-term smoke exposure studies. The mixed fuel cribs generate repeatable fire smoke to further investigate the evolution of structure fire smoke including chemical and aerosol transport and exposure concentration downwind, aerosol dynamics and deposition, aging and formation of secondary organic aerosols, and off-gassing of semivolatile organic compounds from smoke deposited on surfaces.

## Materials and Methods

### Mixed Fuel Crib

All cribs had a combustible fuel mass fraction of 54.3% ± 0.8%, which is typical of a single-family residence.^1,3^ The cribs all had the same mass fraction of the six materials used. The materials were selected as plausible representations of major components of residential structures. Spruce/pine/fir (SPF) wood (21.6% ± 0.6% by weight) represented framing lumber of a house. The 3/4 in. (19 mm) thick OSB (14.8% ± 0.5%) and 3/4 in. (19 mm) thick type X Gyp (45.7% ± 0.8%) represented additional construction materials. ABS plastic (5.1% ± 0.3%) was chosen as a surrogate for a range of plastic household goods, while PU rubber (5.9% ± 0.3%) represented PU products and foam in upholstered furniture. Other household components, such as pipes, electrical insulation, siding, and flooring, were represented by PVC plastic (7.0% ± 0.4%).

**Figure 1:**
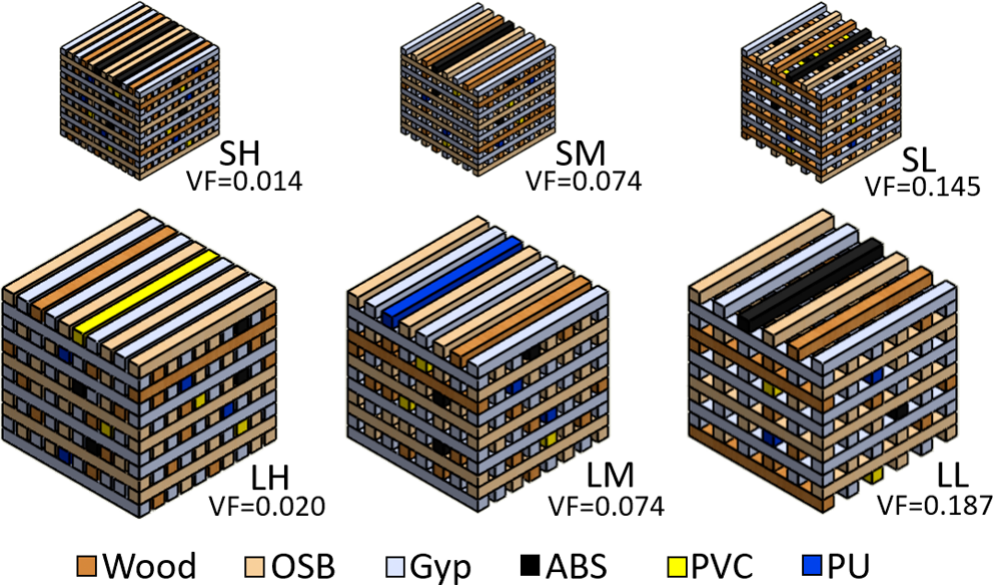
Drawings of the mixed fuel cribs. From the top left, going clockwise: small high, small medium, small low, large low, large medium, and large high-packing density cribs. VF: ventilation factor. Small cribs are 30.0 cm × 30.0 cm × 28.6 cm, and large cribs are 47.5 cm × 47.5 cm × 45.7 cm. Views from other angles are in Supporting Information (SI) Figure 1.

Two crib sizes were constructed with the larger crib configuration (47.5 cm × 47.5 cm × 45.7 cm tall with 3.81 cm thick square sticks) having approximately four times the volume of the smaller crib configuration (30.0 cm × 30.0 cm × 28.6 cm tall with 1.91 cm thick square sticks). The large and small cribs were divided into subgroups of three packing densities: low, medium, and high, each with varying stick counts per layer and fixed gap spacing between the sticks (Figure 1 and SI Table 1). Ventilation factor, Ψ, based on the crib construction, was used to characterize flaming combustion of the cribs.^9^ Ventilation factor is calculated from eq 1 using the stick thickness, *b*, the height of the crib, *h*, and the ratio of the open area of the vertical shafts, *A*_*v*_, to the exposed surface area of the fuel sticks, *A*_*s*_.
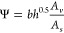
1

**Table 1:** Burning and Emission Properties of Mixed Fuel Cribs^a^

Crib type	Burn time (min)	Total mass loss (kg)	THR (MJ)	Peak HRR (kW)	MCE	Avg. effective heat of combustion (MJ/kg)
SL	30 ± 0	4.39 ± 0.06	67.5 ± 1.1	130 ± 18	0.970 ± 0.001	15.4 ± 0.2
SM	45 ± 0	5.83 ± 0.06	89.2 ± 2.4	79 ± 17	0.971 ± 0.002	15.3 ± 0.3
SH	127 ± 8	8.24 ± 0.29	114 ± 4	24 ± 1	0.953 ± 0.001	13.8 ± 0.3
LL	45 ± 0	20.9 ± 0.3	315 ± 3	279 ± 15	0.975 ± 0.001	15.1 ± 0.1
LM	90 ± 0	29.5 ± 0.3	446 ± 4	220 ± 15	0.970 ± 0.002	15.1 ± 0.2
LH	190 ± 8	37.1 ± 0.6	498 ± 17	91 ± 23	0.955 ± 0.005	13.4 ± 0.5

a Mean values and combined uncertainty with a coverage factor of two for repeated experiments reported.

The experimental campaign burned 21 cribs: four small and three large cribs of each packing density. Sticks were glued together by wood glue consisting of polyvinyl acetate. Since the glue weighs less than 0.7% of the total weight, the fuel loading and mass of the glue were assumed to be negligible. The small cribs were glued to a Gyp base plate weighing 1.38 kg ± 0.05 kg to add stability.

The cribs were conditioned in the same room as the test facility, which maintained a temperature of 26 °C ± 2 °C and relative humidity of 44% ± 6%. The moisture content of the SPF wood, OSB, and Gyp was measured by heating preweighed samples in an oven at 105 °C for 24 h, then weighed to obtain an estimate of the free water moisture, representing dry fuel mass. From the mass readings before and after heating, the water content of the three materials was calculated; the moisture content of SPF wood is 9.9% in weight, 7.9% in weight of OSB, and 16.9% in weight for Gyp. The calcium sulfate dihydrate in the Gyp contains approximately 21% chemically bound water by mass and some of the moisture loss measured is presumed to be from dehydration.

### Fire Behavior and Combustion Product Yield Measurements

All burns were conducted using a 0.5 MW calorimeter (SI Figure 2). For each experiment, cribs were placed on a load cell (Mettler-Toledo GmbH, PBK 989-B60^a^) to measure the mass loss while burning. Two load cells were implemented for experiments burning large cribs with either medium or high packing density, accounting for the higher crib weight, which exceeded a single load cell capacity (60 kg). The fire plume was captured by the calorimeter’s exhaust hood, and samples were continuously pulled from the exhaust duct at a well-mixed location (approximately 50 duct diameters downstream of the hood inlet) to provide measurements of heat release, particulate smoke, and gas phase emissions generated by the fire. HRR was measured using the principle of oxygen consumption calorimetry.^11^ Bryant et al.^11−13^ provide details of the calorimeter and the associated exhaust gas analysis and exhaust flow measurement used to determine HRR. To account for the different fuel loadings and the scale of emissions, the exhaust flow was set at nominally 2.1 kg/s of dry air for the small cribs and 3.0 kg/s of dry air for the large cribs. Environmental conditions such as temperature, relative humidity, and pressure in the facility were continuously monitored.

Species concentrations used to estimate yields of carbon dioxide (CO_2_) and CO were measured using the calorimeter’s nondispersive infrared (NDIR) gas analyzer (Siemens, Ultramat 6E) as described in Bryant and Bundy.^11^ HCN, acrolein (C_3_H_4_O), formaldehyde, NO_*x*_, sulfur dioxide (SO_2_), and total hydrocarbon (THC) concentrations were measured using a Fourier transform infrared (FTIR) gas analyzer (Thermo Scientific, Antaris IGS FTIR Gas Analyzer).^14^ The FTIR implemented a 10 m path length cell (2.0 L in cell volume) operating at a 4 cm^–1^ spectral resolution. Gas species measurements were obtained using a custom-built analytical method provided by the manufacturer. The method included calibration for each species ranging from 10 to 1000 ppm except for CO_2_ as high as 200,000 ppm and CO as high as 100,000 ppm. Calibrations were verified daily using midrange gas standards comprised of every species of interest. Background signals were minimized daily via a N_2_ purge gas. THC concentration is the sum of methane, acetylene, ethene, ethane, propylene, propane, isobutene, and *n*-pentane concentrations. Samples flowed into the FTIR at approximately 5 Lpm, indicating a 24 s residence time within the gas cell. Since the HRR and NDIR sensors were recorded at 1 Hz, the FTIR data was averaged over the duration of residence time to synchronize concentration measurements.

Real-time smoke concentrations were measured using a smoke concentration meter.^15^ The smoke concentration meter, consisting of a He–Ne laser going across the exhaust duct with a Silicon amplified photodetector on the other end, takes the ratio of the incident initial, clean air signal and the smoke-filled duct signal to quantify smoke concentration. Mulholland et al.^15^ describe the setup in more detail.

The light attenuation of a monochromatic light beam through a fixed path length is called extinction, eq 2 shows the relationship between the ratio of intensities of the incident light (*I*_0_) to transmitted light (*I*), light path length (*L*), and the light extinction coefficient (*K*),
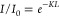
2where *K* and smoke mass concentration (*C*_*m*_) are related through a mass-specific extinction coefficient (*K*_*m*_) by eq 3.
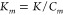
3*K*_*m*_ depends on the smoke size distribution and the optical properties of the smoke, and the average for smoke from flaming combustion of various fuels was applied: 8.7 m^2^/g for a 633 nm wavelength light source.^16^

The smoke production rate, *SPR* (m^2^/s), is calculated by eq 4 where *K* (m^–1^) is multiplied by the duct volumetric flow (*V̇*, in m^3^/s).
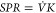
4While *SPR* is time specific, *SPR* integrated over time is the total smoke production in units of m^2^.

A specific extinction area, *SEA* (m^2^/kg), is the quotient of *SPR* and the fuel mass loss rate (*ṁ*_*fuel*_) shown in eq 5:
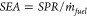
5

The smoke yield was determined from gravimetric smoke measurements using Teflon particulate filters. A stainless-steel sampling probe was inserted in the middle of the duct for filter collection and sampling of volatile organic compounds (the results of which will be presented in a subsequent publication). A stainless-steel filter holder containing a 47.0 mm Teflon filter (selected for its hydrophobicity) pulled effluent smoke sample at 2.5 Lpm by a data-logging pump (Sensidyne, Gilair Plus). The pump was equipped with a Drierite filter to monitor dry air flow. A bypass pump adjusted the flow to meet isokinetic sampling conditions; the total flow through the probe was set at 12.93 Lpm ± 0.29 Lpm for the small cribs and 25.06 Lpm ± 0.22 Lpm for the large cribs. A heating tape was wrapped around the filter holder for all the large crib burns to avoid condensation on the filter. The filter samples were collected for the test duration for all other experiments. The Teflon filters were conditioned in a desiccator for at least 24 h before weighing with a microbalance (Sartorius, 98648–04–81).

The mass emission rate (*ṁ*) of a chemical species at time, *t*, was calculated using eq 6:
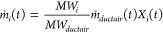
6where *i* is the chemical species of interest, *MW* is the molecular weight, *ṁ*_*duct air*_ is the mass (dry) air flow of the duct, and *X* is the volume fraction of species *i*, background corrected. Fire-integrated yield (*Y*_*i*_), also known as emission factor, is the sum of the mass emission rate throughout the burn from ignition (*t = ign*) to the end of sampling (*t = end*) divided by the total fuel mass loss (eq 7).
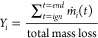
7The smoke yield (*Y*_*smoke*_) is calculated using eq 8:
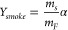
8where *m*_*s*_ is the mass of the smoke collected on the filter, *m*_*F*_ is the total mass loss of the sample, and α is the ratio of the mass flow of air through the exhaust duct to the mass flow through the filter sampler.

An integrated modified combustion efficiency (*MCE*)^17,18^ was calculated using eq 9, which is the ratio of the sum of the CO_2_ moles generated from the burn, *n*_*ĊO*_2__, over the sum of CO_2_ and CO moles, *n*_*ĊO*_, generated from the burn.
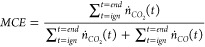
9MCE is often used to characterize the phase of flaming and smoldering combustion in wildland fuels, and the yields of many compounds have been shown to correlate with MCE.^18−20^ The average effective heat of combustion for the mixed fuel package was determined from the quotient of the total heat release measured from the calorimetry measurement system and the total mass loss. The combined uncertainties of reported values are in SI Table 3.

### Test Procedure

Each experiment started with loading the filter holder with a new Teflon filter and collecting background for the calorimeter and other online instruments (FTIR, gas analyzer, and smoke extinction). Before zeroing the load cell, three layers of insulating ceramic fiber boards were placed on the load cell, then either a metal tray or a gypsum wallboard covered with aluminum foil and ceramic paper on top. Gel fire starters (consisting of paraffin and other hydrocarbons, weighing 50.9 g ± 0.6 g) were placed on top of a crib (number of gel packs used in SI Table 1). Filter sampling started when all gel packs ignited, indicating the start of a test (time = 0 s). Cribs were burned until reaching a minimum of 55% (typically 65%) mass loss when HRR reached back to background level. The smoke filter sampling ended about a minute before the experiment ended.

## Results and Discussion

### Combustion Characteristics

**Figure 2:**
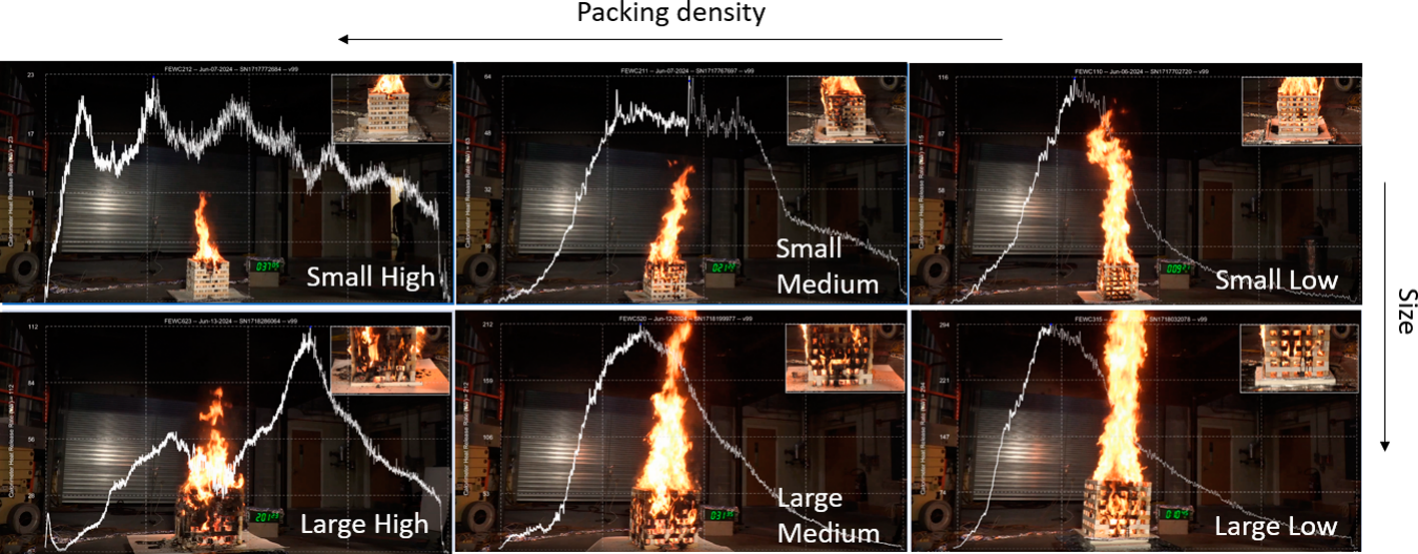
Snapshots of each crib type at the time of the peak HRR, zoomed into the crib in the top right window, overlaid with HRR plot over time (these HRR plots are also in SI Figure 3).

Once ignited, gel packs accelerated the fire spread from the top of the crib downward toward the center of the crib. The fire developed differently for the six different crib types; the differences observed are discussed using Table 1, Figure 2, and SI Figure 3. The low-packing density cribs burned more intensely with the fire quickly reaching the peak HRR, within 15 min from ignition for the small crib and by 30 min for the large crib. The high packing density cribs did not have a peak HRR as high as the low packing density cribs but instead hovered at a lower HRR for a longer period. While the plume height was not measured, one can visually see in Figure 2 that the plume was weaker for the high-packing density cribs; the plume is more than twice the height of the crib itself for the medium and low-packing density cribs but not for the high-packing density cribs.

The total heat release (THR) and the time it takes to burn combustible fuel in the crib (burn time) increased as the packing density and fuel load increased; however, the peak HRR decreased with an increase in packing density (Table 1). The HRR profiles (SI Figure 3a and b) show that a low-packing density crib with a higher ventilation factor has a higher peak HRR but burns faster (approximately 30 to 45 min). The high-packing density crib with a lower ventilation factor has a low peak HRR but takes longer to burn (up to 3.25 h). The smaller fire size for cribs with higher packing density suggests limited ventilation to the pyrolysate, reducing combustion. Furthermore, the peak HRR of the large cribs is more than double that of the small cribs with similar packing density. The time to reach the peak HRR is inversely related to the ventilation factor. The scale of the cribs also affected the duration of the burn; for the medium-packing density cribs with similar ventilation factors at 0.07 cm, the large medium-packing density crib (about five times higher in mass loss) took twice as long to burn compared to the small medium-packing density crib.

A similar behavior as HRR was observed for mass loss rate where the total mass loss increases with increasing packing density but the peak mass loss rate decreases (SI Figure 3c and d). The peak mass loss rate is higher for low-packing density/high-ventilation cribs, and the time to reach the peak mass loss rate is correlated with packing density. The peak mass loss rate doubled for the large cribs compared to the small cribs with similar ventilation factors; for example, the peak mass loss rate reached 6 g/s for the small medium-packing density crib compared to 14 g/s for the large medium-packing density crib in SI Figure 3.

Table 1 also provides the estimated average effective heat of combustion of the mixed fuel crib and its MCE. The average effective heat of combustion for the mixed fuel cribs ranges from 13.4 MJ/kg to 15.4 MJ/kg, which is in between the heat of combustion for wood^21^ and plastics.^22^ The average effective heat of combustion for the cribs with medium and low packing densities is around 15.2 MJ/kg; however, the average effective heat of combustion is lower for high-packing density cribs with corresponding lower ventilation factors and MCEs with a mean value of 13.6 MJ/kg (SI Figure 4). The observed decrease in the effective heat of combustion for the high packing density cribs is likely due to the observed reduction in combustion efficiency and possibly an increase in the dehydration of gypsum over the extended burning time, leading to increased mass loss of the residue with no corresponding oxygen consumption.

**Figure 3:**
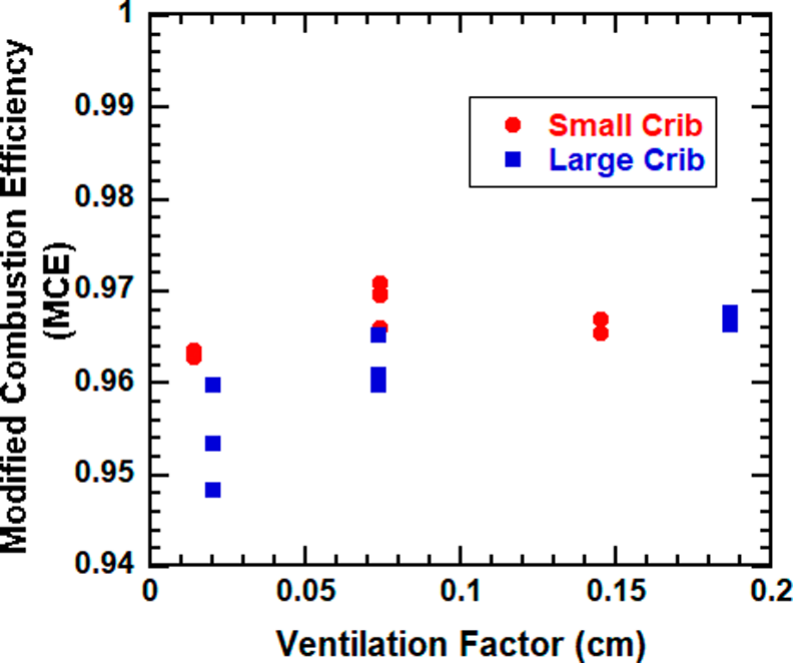
MCE versus ventilation factor for large (square) and small (circle) cribs. Mean values and combined uncertainty with a coverage factor of 2 for repeated experiments reported.

Real-time MCE fluctuated between 0.83 and 1.00 throughout the burn (SI Figure 5) as the flame traveled from the top of the crib to the bottom. When integrating across the burn event, the MCE decreased for the high packing density cribs for both crib sizes (Table 1). This trend in MCE is more observant with the large cribs. The correlation between crib packing density, MCE, and ventilation factor is also observed in Figure 3; MCE and the ventilation factor tend to decrease for the cribs with high packing density. The large range in MCE within the crib types, especially for the large cribs, shows the large test-to-test variability for an open flame calorimetry experiment.

### Species Yields

Tables 2 and 3 provide the yields of smoke and gas species.

**Table 2:** Yields of Mixed Fuel Cribs^a^

Crib type	Smoke (g/kg)	Specific extinction area (m^2^/kg)	CO_2_ (g/kg)	CO (g/kg)
SL	15.5 ± 0.3	123 ± 9	1389 ± 8	42.9 ± 1.7
SM	13.3 ± 0.7	101 ± 5	1372 ± 22	40.7 ± 2.3
SH	14.2 ± 0.8	94 ± 6	1216 ± 25	60.1 ± 1.0
LL	13.9 ± 2.0	94 ± 2	1390 ± 9	35.9 ± 1.0
LM	12.8 ± 4.4	108 ± 12	1374 ± 22	42.5 ± 2.8
LH	15.6 ± 4.9	119 ± 19	1176 ± 47	55.7 ± 4.3

a Smoke yield is derived from gravimetric measurements, specific extinction area from the duct smoke meter, and CO_2_ and CO from NDIR measurements. Mean values and combined uncertainty with a coverage factor of two for repeated experiments reported.

**Table 3:** Yields of Mixed Fuel Cribs^a^

Crib type	THC (g/kg)	NO_*x*_ (g/kg)	HCN (g/kg)	SO_2_ (g/kg)	C_3_H_4_O (g/kg)	HCHO (g/kg)
SL	2.9 ± 1.3	2.1 ± 0.5	0.56 ± 0.22	2.5 ± 1.2	0.51 ± 0.38	0.18 ± 0.11
SM	2.9 ± 0.2	2.1 ± 0.5	0.37 ± 0.07	7.9 ± 1.4	0.52 ± 0.22	0.08 ± 0.09
SH	7.7 ± 0.8	2.2 ± 0.3	0.32 ± 0.10	10 ± 1.6	0.60 ± 0.41	0.24 ± 0.13
LL	2.1 ± 0.3	2.1 ± 0.3	0.21 ± 0.15	6.5 ± 0.56	0.49 ± 0.23	0.06 ± 0.09
LM	2.9 ± 1.3	1.0 ± 0.4	0.36 ± 0.10	12 ± 4.2	0.64 ± 0.38	0.20 ± 0.10
LH	6.3 ± 1.8	0.92 ± 0.45	0.24 ± 0.08	15 ± 4.6	0.74 ± 0.50	0.31 ± 0.13

a Total hydrocarbon (THC), nitrogen oxides (NO_*x*_), hydrogen cyanide (HCN), sulfur dioxide (SO_2_), acrolein (C_3_H_4_O), and formaldehyde (HCHO) from the FTIR measurements. Mean values and combined uncertainty with a coverage factor of two for repeated experiments reported.

### Carbon Dioxide and Carbon Monoxide

**Figure 4:**
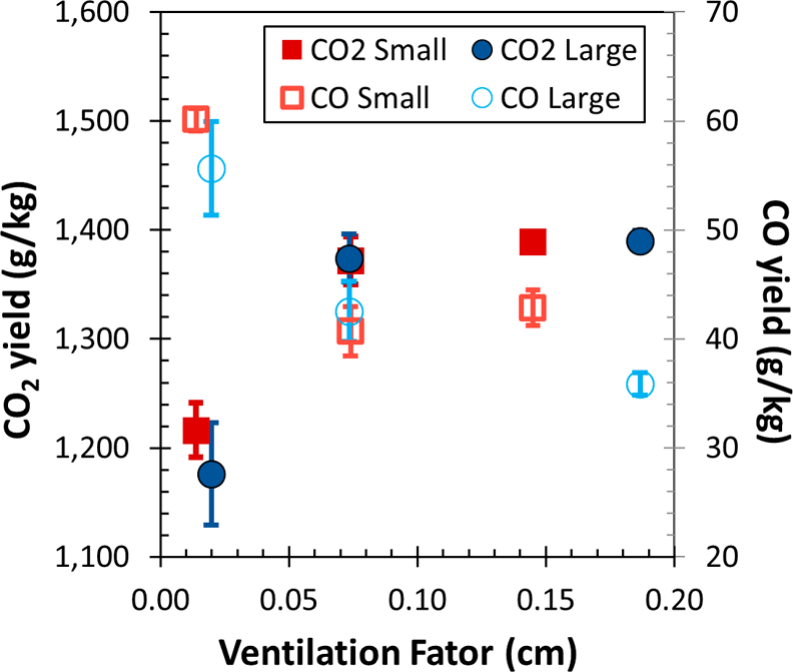
CO_2_ (left axis, solid) and CO (right axis, hollow) yields over crib ventilation factors for large (circle) and small (square) cribs. Mean values and combined uncertainty with a coverage factor of 2 for repeated experiments reported.

The CO_2_ yield ranges between 1176 g/kg and 1390 g/kg of fuel, whereas the CO yield ranges from 35.9 g/kg to 60.1 g/kg of fuel. Figure 4 shows the inverse relationship between CO_2_ and CO yields, the high packing density cribs with a ventilation factor less than 0.02 cm show a more significant increase in CO yield and a decrease in CO_2_ yield, while the yield values are comparable (within the standard deviation of one another) for the medium and low-packing density cribs with ventilation factors greater than 0.07 cm. The difference between the yield trends of CO_2_ and CO can be attributed to oxygen availability, which, if limited to the fuel source, can result in incomplete combustion products (i.e., CO). Since the small and large crib results overlap for both CO and CO_2_ yields in Figure 4, the size and fuel mass do not affect the yields as much as the packing density/ventilation factor does. The carbon mass fraction of the dry mixed fuel crib is 66%, higher than the biomass fraction typically used^17,19,20,23^ (more in SI).

### Smoke

**Figure 5:**
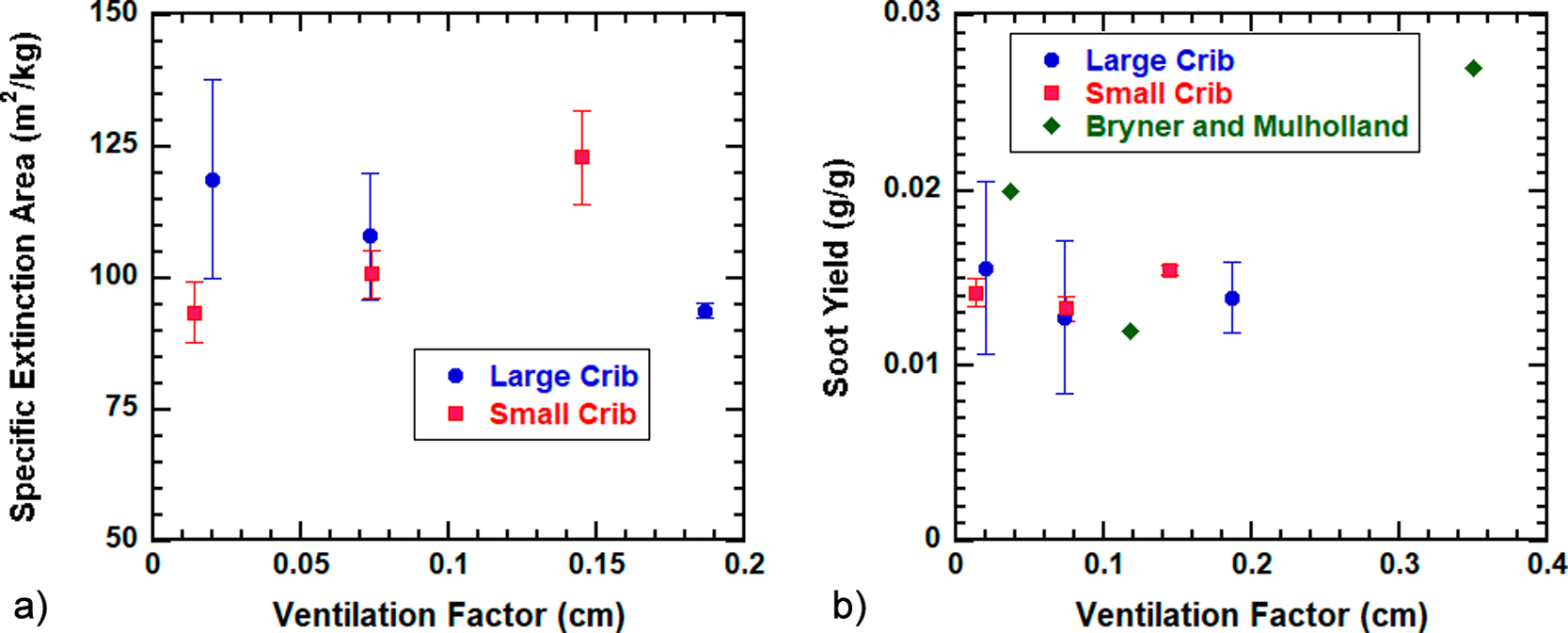
Specific extinction area (a) and gravimetric smoke yields (b) over crib ventilation factors for large (red) and small (blue) cribs. Mean values and combined uncertainty with a coverage factor of 2 for repeated experiments reported. Smoke yields presented in Bryner and Mulholland^9^ are also shown in triangles.

The smoke emission profile is also like HRR, reaching the peak smoke production rate at the same time as the peak HRR and mass loss rate (SI Figure 7). The smoke extinction meter and gravimetric smoke measurement both do not show their dependency on the crib scale since the yields overlap for the two sizes (Figure 5). The gravimetric smoke yield ranges from 0.0079 g/g fuel to 0.0196 g/g fuel, showing little variation across both sets of cribs. Similar results were found by Bryner and Mulholland where scale did not affect the smoke yield, however, they found that individual components of mixed fuel (like plastic) can have a bigger effect on smoke yield than the ventilation factor.^9^ Smoke yields of a mixed fuel can be estimated by mass-weighting to that of each component,^9^ and here, the mass ratio of all fuel types remains the same across all cribs. Therefore, smoke yield is expected to remain the same. Sooting is expected to be higher with lower/inadequate oxygen availability for complete combustion, and this may have been the case for the large cribs where the total smoke extinction per mass burned increased as the packing density increased. However, this was not the case for the small cribs. While the differences may seem insignificant, the minimum smoke yield was observed near the ventilation factor of 0.1 which is in agreement with Bryner and Mulholland.^9^

### Trace Gases

**Figure 6:**
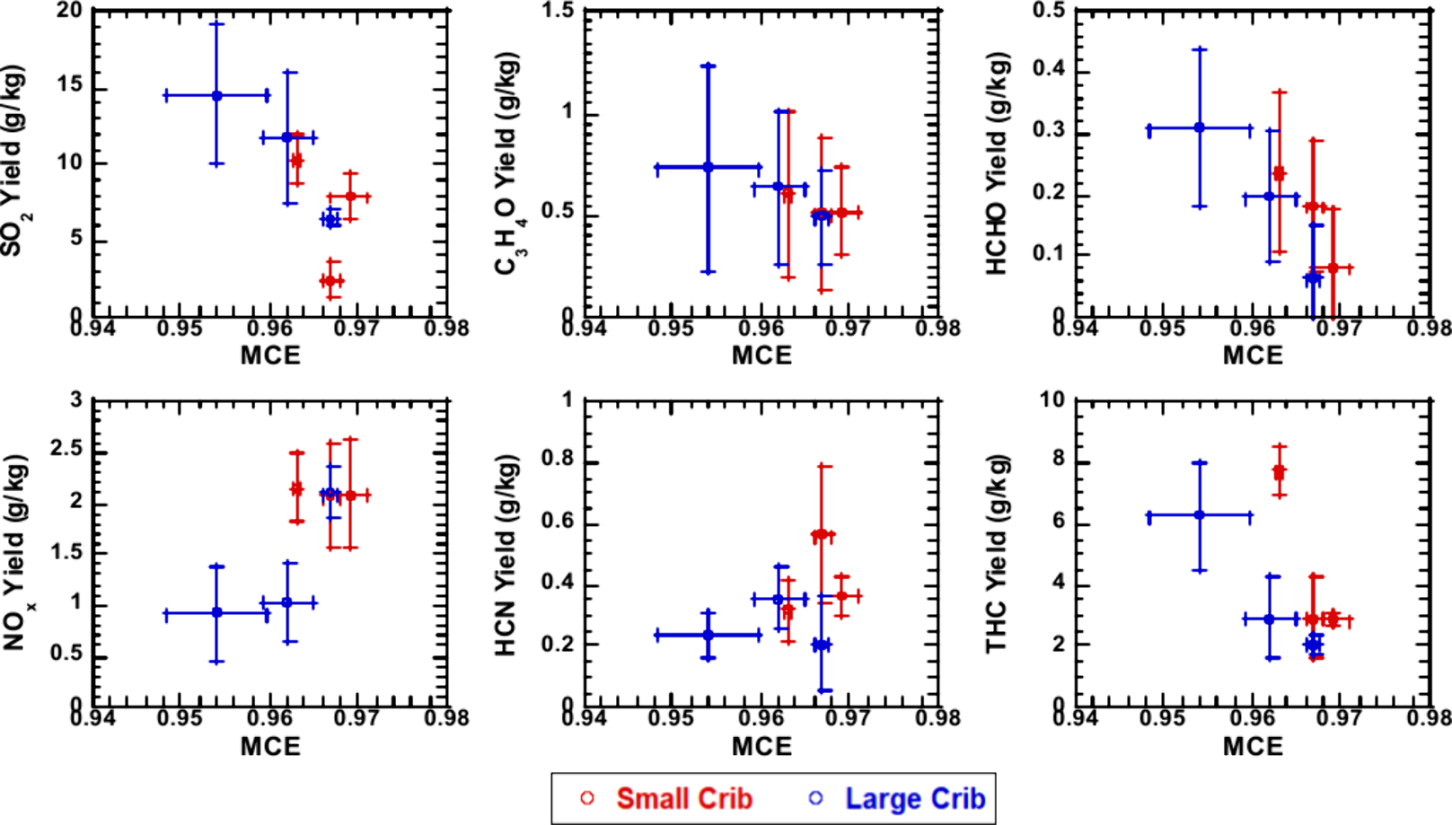
Trace gas yields against MCE for large (blue) and small (red) cribs. Mean values and combined uncertainty with a coverage factor of 2 for repeated experiments reported.

The THC, NO_*x*_, and SO_2_ yields from the mixed fuel crib burn are an order of magnitude higher than the yields of HCN, acrolein, and formaldehyde (Table 3). Emission yields of trace gases from biomass burns have been well correlated with MCEs.^18−20,23^ However, a wide range of MCEs was observed here for the same construction of mixed fuel cribs. The trace gas yields obtained from the FTIR are compared against both MCEs in Figure 6 and the ventilation factors in SI Figure 8.

The average yields of hydrocarbon trace gases (acrolein, formaldehyde, and THC) and SO_2_ correlated with both MCE and ventilation factors; the yields tend to increase with increasing packing density and decreasing MCE and ventilation factor similar to CO, all of which are linked to incomplete combustion. For SO_2_ and THC, the yields doubled with the differences in packing density and 0.02 difference in MCE. SO_2_ yields are higher for lower ventilation factors (SI Figure 8), but this may be skewed since lower ventilation tests typically lasting longer, therefore having more time to heat Gyp (which contains sulfur). Despite the standard deviations for acrolein and formaldehyde being larger than the mean yields, a slight decrease in the yields with increasing ventilation and MCE is observed. A clear trend was not observed for NO_*x*_ and HCN. Thermal NO_*x*_ production is dependent on flaming temperature and residence time.^24^ Here, the two factors, heat relase and cumulative residence time, may have canceled each other out with low packing density cribs having higher peak HRR but shorter burn time and higher packing density cribs having lower peak HRR but longer burn time, resulting in similar NO_*x*_ yields for all crib types. While HCN is a combustion product of ABS^25^ and PU,^26^ the nitrogen mass fraction remaining constant across the cribs which may have contributed to the yields remaining similar across the ventilation factor and MCE.

**Figure 7:**
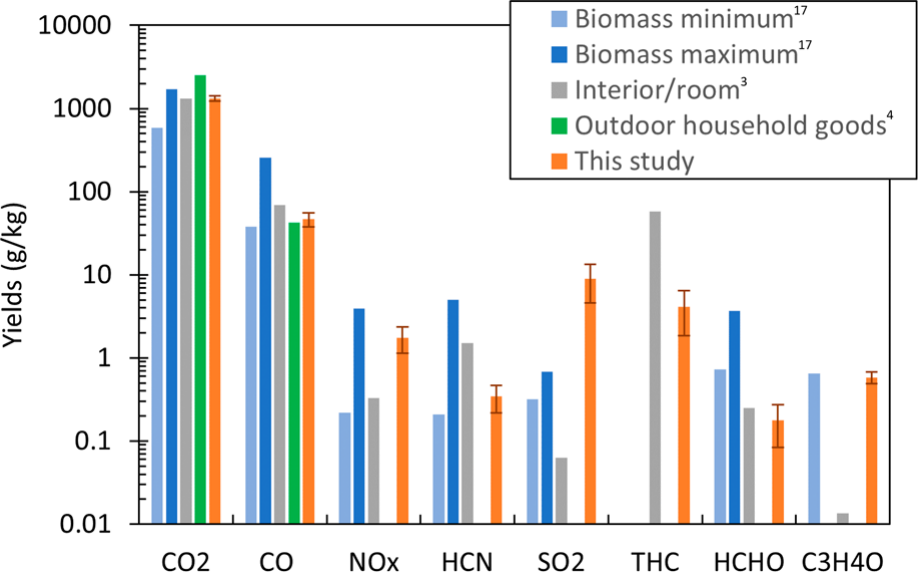
Emission yields of CO_2_, CO, NO_*x*_, HCN, SO_2_, THC, HCHO, and C_3_H_4_O against other literature. Biomass minimum and maximum yields are from Akagi et al.,^17^ interior/room burn yields are from Holder et al.,^3^ and the yields of outdoor household goods are from Vacca et al.^4^ Biomass minimum for acrolein is the average value for biomass. Error bars not in log scale.

The yields reported here are comparable to those collected from biomass burns,^17^ interior/room burns,^3^ and burns of outdoor furniture and goods^4^ (Figure 7). CO and CO_2_ yields obtained from the mixed fuel cribs are within the range of biomass yields and are comparable to room and outdoor goods yields. NO_*x*_ and HCN yields from the cribs are in the range of yields from biomass burns. NO_*x*_ yield is higher than the yield for room burns, and HCN yield is lower than that for room burns. HCN may be lower due to room burn fuels having higher nitrogen ratio (such as PU foam products) and/or the oxygen availability during the experiments; the cribs were burned in well ventilated open space whereas the room burns in Holder et al.^3^ may have been under ventilated. O_2_ increases the formation of radicals removing HCN,^27^ therefore HCN yield is known to increase as ventilation decreases.^25^ Acrolein yield from the cribs is comparable to the yield from biomass. The formaldehyde yield from the mixed fuel cribs is comparable to the yield from room burns.

SO_2_ yields from the cribs are higher than those obtained from biomass and room burns which do not have a large mass fraction of Gyp in their fuel as the cribs do. SO_*x*_ are emitted only if their precursors are in the fuel consumed,^1^ therefore, SO_2_ may have the potential to be used as a chemical fingerprint for WUI structure fires.

## Implications

The mixed fuel cribs studied here showed how HRR, the average effective heat of combustion, duration of the burn, and the time it takes to reach the peaks of mass loss rate and effluent concentrations can fluctuate based on scale and packing density despite the six crib types having the same mass fractions of wood, gypsum, and plastics. A crib’s packing density and corresponding ventilation factor drove the differences observed in burn duration, peak HRR, peak mass loss rate, MCE, and smoke emission rates and yields. The differences in the studied packing densities (ventilation factors ranging from 0.014 to 0.187) resulted in differences in the yields of CO_2_ by 16%, CO by 52%, NO_*x*_ by 74%, HCN by 102%, SO_2_ by 139%, total hydrocarbons by 135%, formaldehyde by 140%, acrolein by 43% and smoke by 20%. These findings support the complexity of the relationship between the fire from mixed fuel sources and the effluent smoke emissions and the importance of packing density when characterizing structure fires.

Most of the studied mixed fuel crib yields seem to fall between vegetation and manufactured materials but differences should be highlighted. HCN, THC, and formaldehyde yields from the mixed fuel cribs are lower than those from the compartment room fire studies, indicating that mixed fuel burns such as WUI/structure fire emission cannot be estimated from compartment fire yields where oxygen may be limited. On the other hand, NO_*x*_ and SO_2_ yields may be higher for structure fires as their combustion characteristics will be different and the fuel load will likely have higher synthetic materials and Gyp which has high sulfur content. The higher carbon mass fraction of dry fuel of the mixed fuel cribs should also be noted, if WUI fire fuel loading is being estimated from the carbon mass balance method then the applied carbon mass fraction should be higher than what is typically used for biomass fuel, 50%.

The burning behavior of mixed fuel fires found here could help study and predict fire spread within WUI communities. These findings can also be applied to building designs, for example, reducing smoke emissions from a structure fire by decreasing the use of plastics. To our knowledge, the emission yields found here are one of the first to capture mixed fuel fire emissions using an oxygen consumption calorimetry system of this size. The yields derived from a known amount of fuel could help improve the emissions inventory for WUI fires and the input parameters needed for predicting smoke spread and air quality downwind using an atmospheric transport model. The yields could also be used to understand the intrusion of structure fire smoke species in soil and water near communities affected by WUI fire, which could affect their quality of life long-term.

A repeatable surrogate for structure fire smoke is also needed to study postfire smoke evolution and lasting exposure from settled soot.^5^ The mixed fuel crib presented here provides a foundation for developing an accurate fuel source for generating surrogate structure fire smoke.

## Supporting Information Available

3D drawings and additional information on the cribs, schematic of the test facility, uncertainty analysis, time series plots of HRR, mass loss rate and modified combustion efficiency, smoke production rate, paragraph on carbon mass fraction, average effective heat of combustion against MCE and ventilation factor, and trace gas yields against ventilation factor (PDF)

## Footnotes

a Certain commercial products are identified in this report to specify adequately the equipment used. Such identification does not imply a recommendation by the National Institute of Standards and Technology, nor does it imply that this equipment is the best available for the purpose.
