# Supplementary material for: Burning Characteristics and Smoke Emission from Mixed Fuel Cribs
Source: ACS EST Air. 2025 Mar 21;2(4):540–7. doi: 10.1021/acsestair.4c00275 (PMC11997947; doi:10.1021/acsestair.4c00275)
Supplement: Supplementary file 1 — ea4c00275_si_001.pdf [file ea4c00275_si_001.pdf]

Supporting Information

Burning characteristics and smoke emission from mixed fuel cribs

By Aika Y. Davis, Thomas G. Cleary, Ryan L. Falkenstein-Smith, Rodney A. Bryant

Materials and Methods

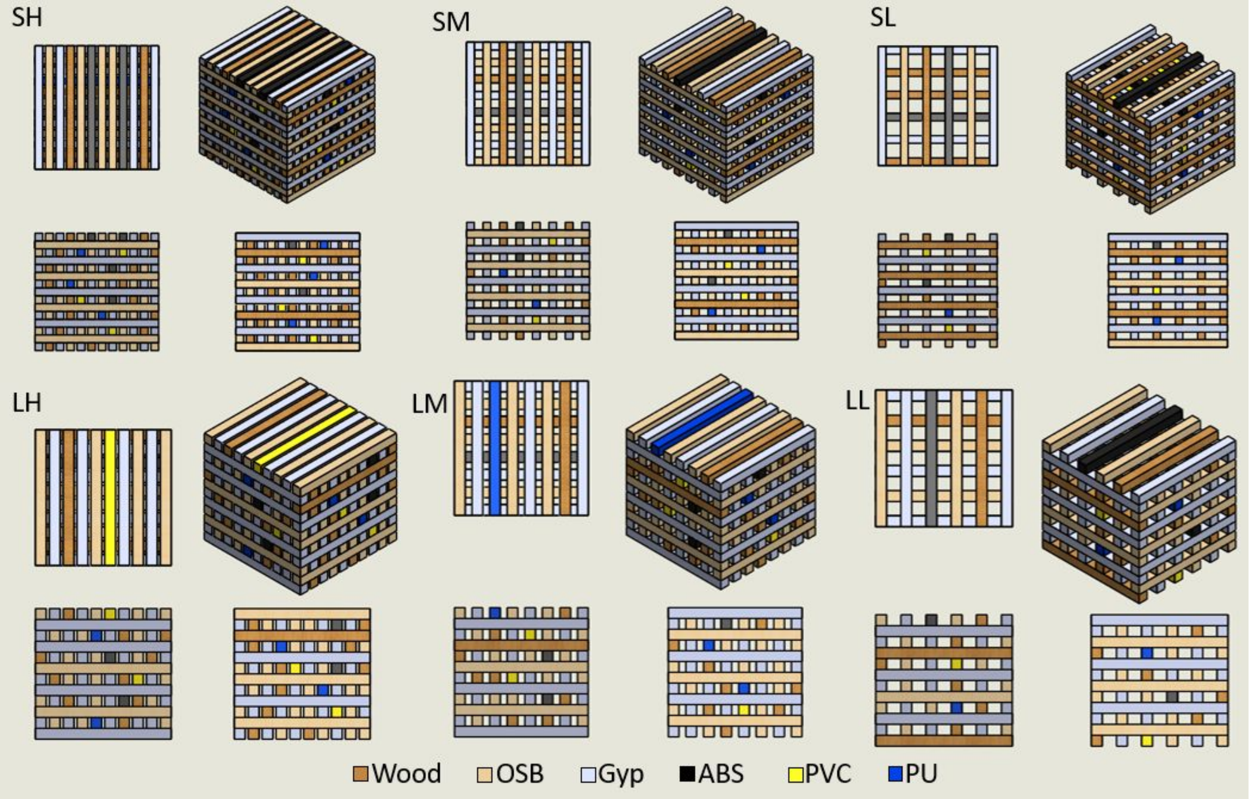

SI Figure 1: 3D drawings of the 6 crib types: top, 3D, left, and right views from top left going clockwise.

SI Table 1: Crib information by crib type. Stick count in SI Table 2.

Size	Packing density	Crib config. (sticks/layer - n layers)	Ventilation factor (cm)	Total sticks	Gel Packs
Small	Low	6-15	0.145	90	2
	Med	8-15	0.074	120	2
	High	12-15	0.014	180	3
Large	Low	6-12	0.187	72	4
	Med	8-12	0.074	96	4
	High	10-12	0.020	120	8

SI Table 2: Crib stick count by crib type.

Size	Packing density	Crib config. (sticks/layer - n layers)	Sticks (count) SPF	OSB	Gyp	ABS	PVC	PU	Total
Small	Low	6-15	30	15	36	3	3	3	90
	Med	8-15	40	20	48	4	4	4	120
	High	12-15	60	30	72	6	6	6	180
Large	Low	6-12	24	12	30	2	2	2	72
	Med	8-12	32	16	39	3	3	3	96
	High	10-12	40	20	48	4	4	4	120

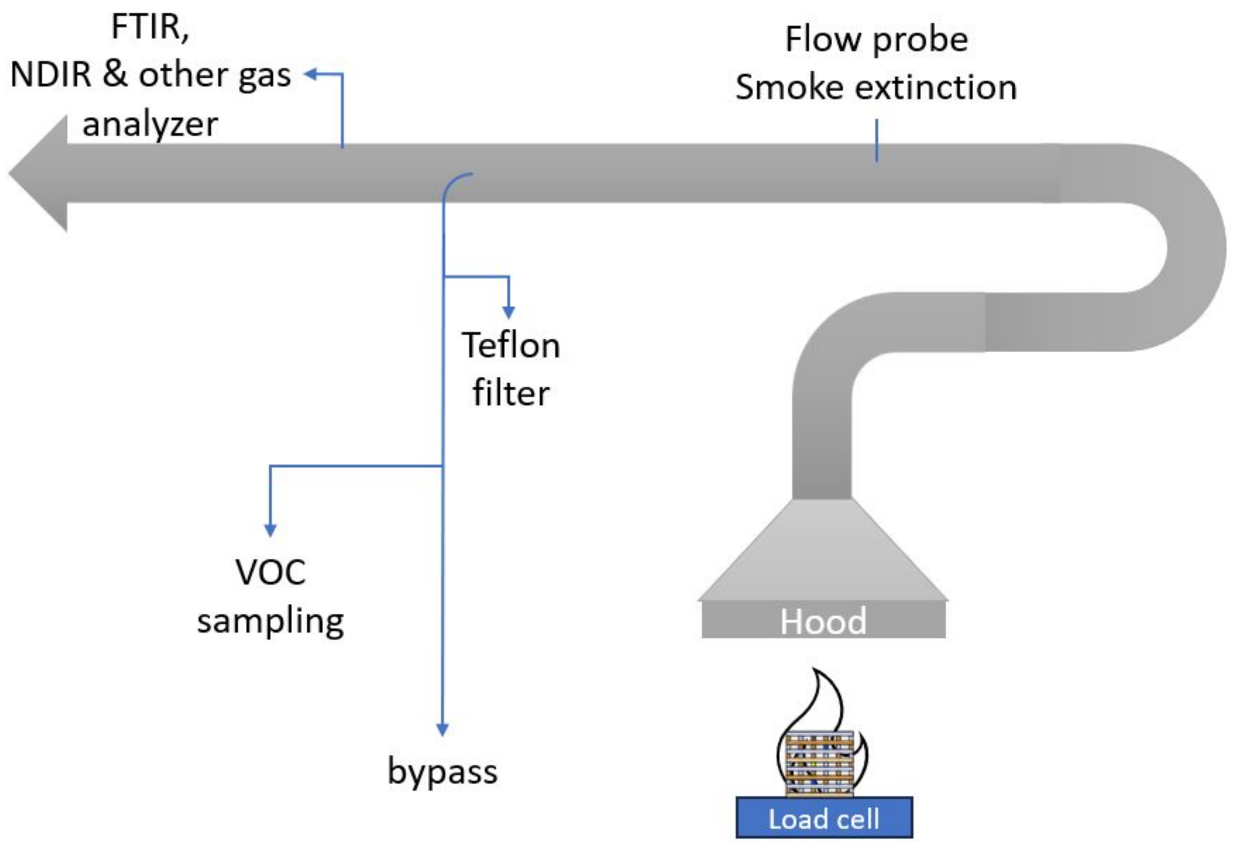

SI Figure 2: The test facility, flow, and measurement setup schematic of the 0.5 MW calorimetry system at the National Institute of Standards and Technology's National Fire Research Laboratory.

Uncertainty Analysis

The uncertainty of heat release and mass loss rates were estimated from the Type B evaluation of uncertainty, defined as the reported instrumentation error. The uncertainty of averaged measurements was determined from the combination of the Type A and B evaluation of uncertainty, where the Type A evaluation of uncertainty is defined by the average variance. The combined uncertainty of calculated parameters discussed in this work, such as modified combustion efficiency, the average effective heat of combustion, and yields of soot and other gas species, were determined from the law of propagation of uncertainty.

SI Table 3: Uncertainty of measurements

Variable (unit)	Combined Relative uncertainty (%)
HRR (kW)	7.9[1]
Avg. effective heat of combustion (MJ/kg)	8.0
Mass loss (kg)	0.1
Total heat release (MJ)	4.0
MCE	0.1
Soot yield (g/kg)	12.0
CO2 yield (g/kg)	2.0
CO yield (g/kg)	1.0
Species yield from FTIR (g/kg)	2.5

Results

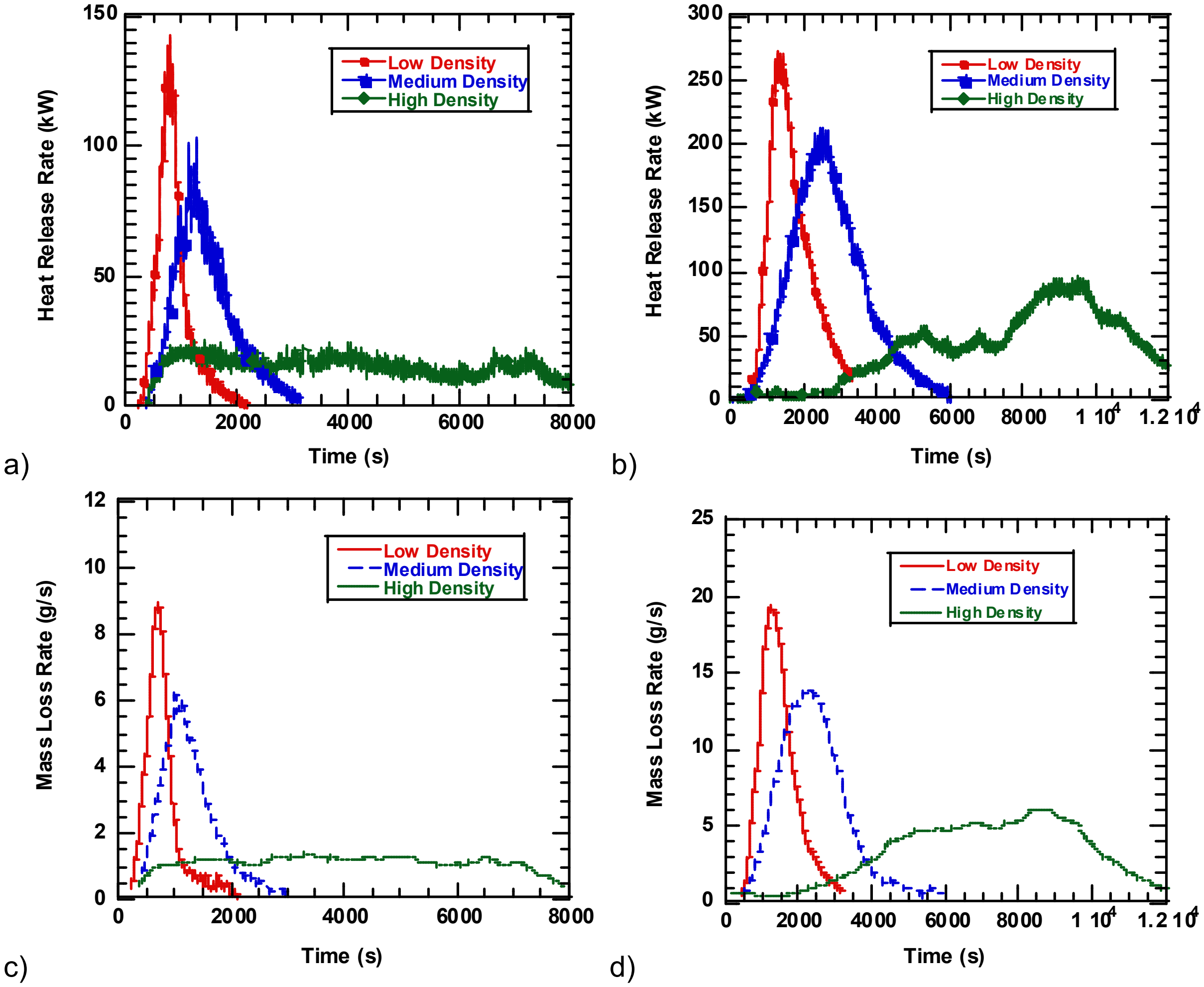

SI Figure 3: HRR profiles (top) and mass loss rate (bottom) of selected tests from each crib type: small cribs (a and c) and large cribs (b and d) with three packing densities.

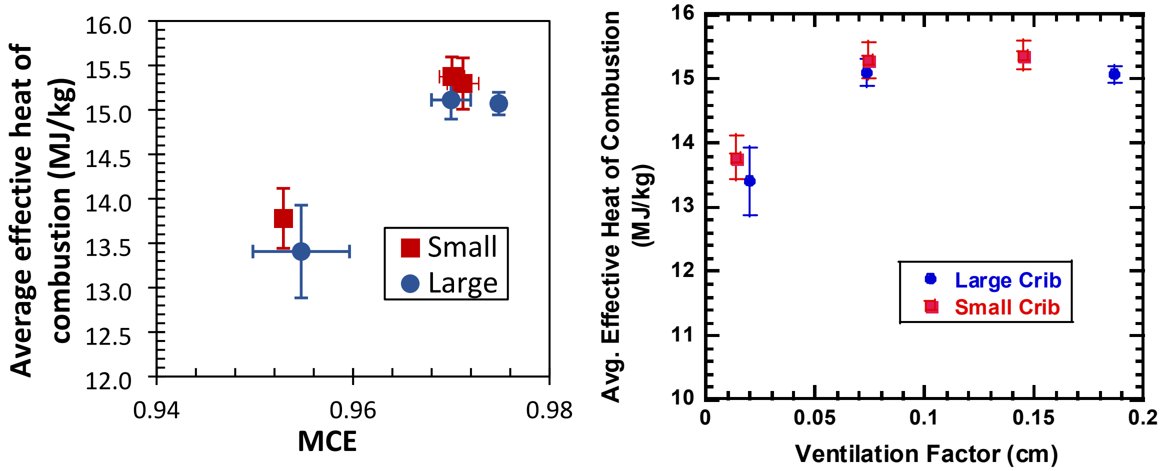

SI Figure 4: Average effective heat of combustion versus MCE (left) for large (circle) and small (square) cribs. Mean values and standard deviation for repeated experiments reported.

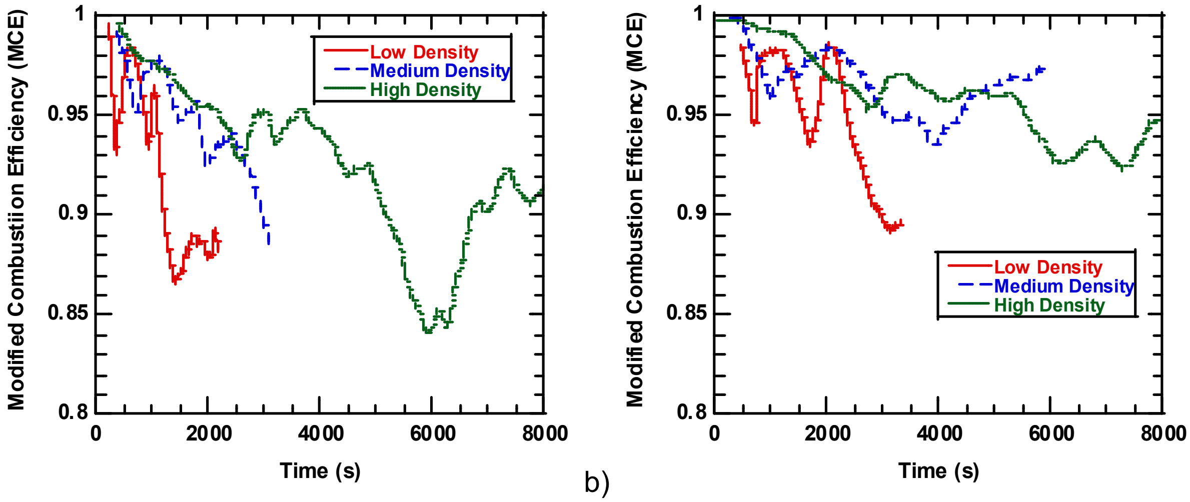

SI Figure 5: Modified combustion efficiency of a selected test from each crib type, small cribs (a) and large cribs (b) with three packing densities.

Carbon mass fraction

The cumulative mass of gas-phase carbon emitted is linearly correlated with the total fuel mass loss (SI Fig. 6). The slope is the carbon mass fraction, which is used to estimate the biomass fuel loading from the carbon mass balance method for biomass burning and wildfires.[2] Often, a 50% carbon content by mass (dry weight) is assumed for the carbon content in biomass fuel.[2–5] From the moisture content measurements using an oven, about 17% of the total mass loss can be attributed to moisture in wood, OSB, and Gyp. Therefore, the carbon mass fraction of the total mixed fuel crib fuel is estimated to be 51% (without adjusting for mass loss due to moisture evaporation from the crib) and the carbon mass fraction of the dry mixed fuel crib is about 66%. This 66% carbon mass fraction indicates that the mixed fuel could have a higher carbon mass fraction of the dry fuel than the biomass fraction by up to 16%.

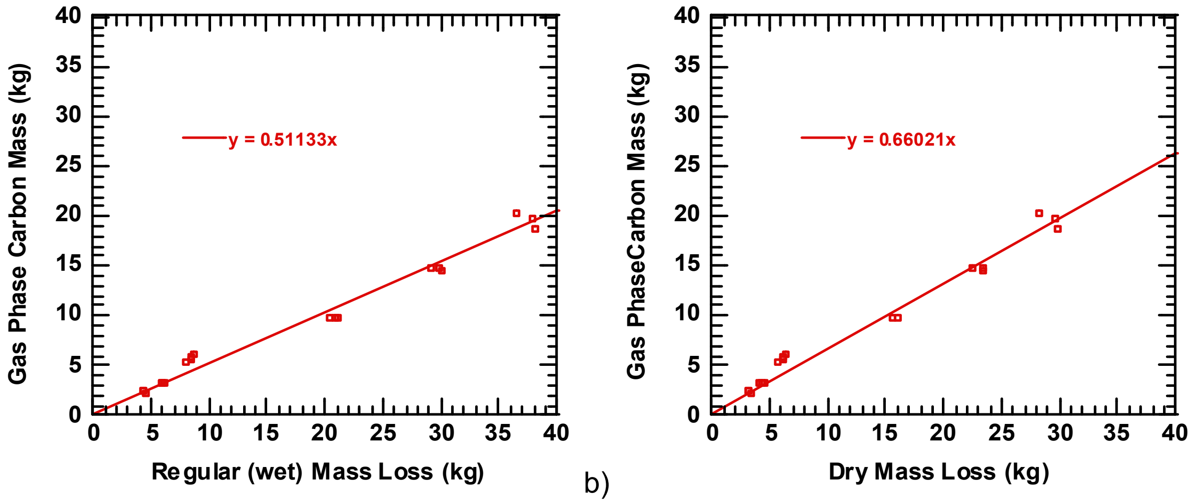

a) b)

SI Figure 6: Total gas-phase carbon mass (from CO_2 and CO) versus mass loss including fuel moisture (a) and without fuel moisture (b). The line represents a best fit through all large and small crib experiments. There may be a crib size-specific linear correlation, potentially due to the construction differences with one being only the small cribs having the gypsum wallboard plate at the bottom.

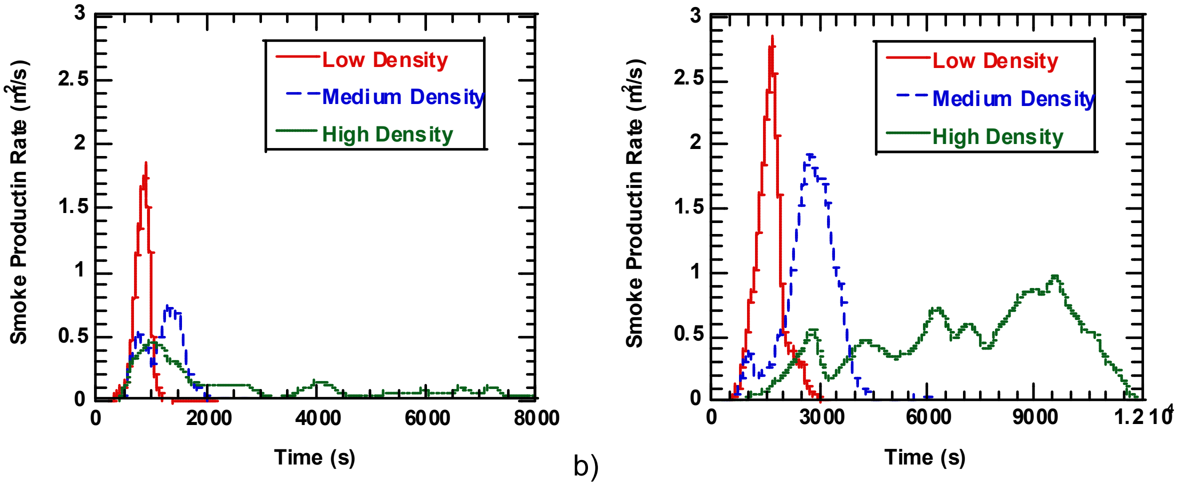

a) b)

SI Figure 7: Smoke production rate of selected tests from each crib type, small cribs (a) and large cribs (b) with three packing densities.

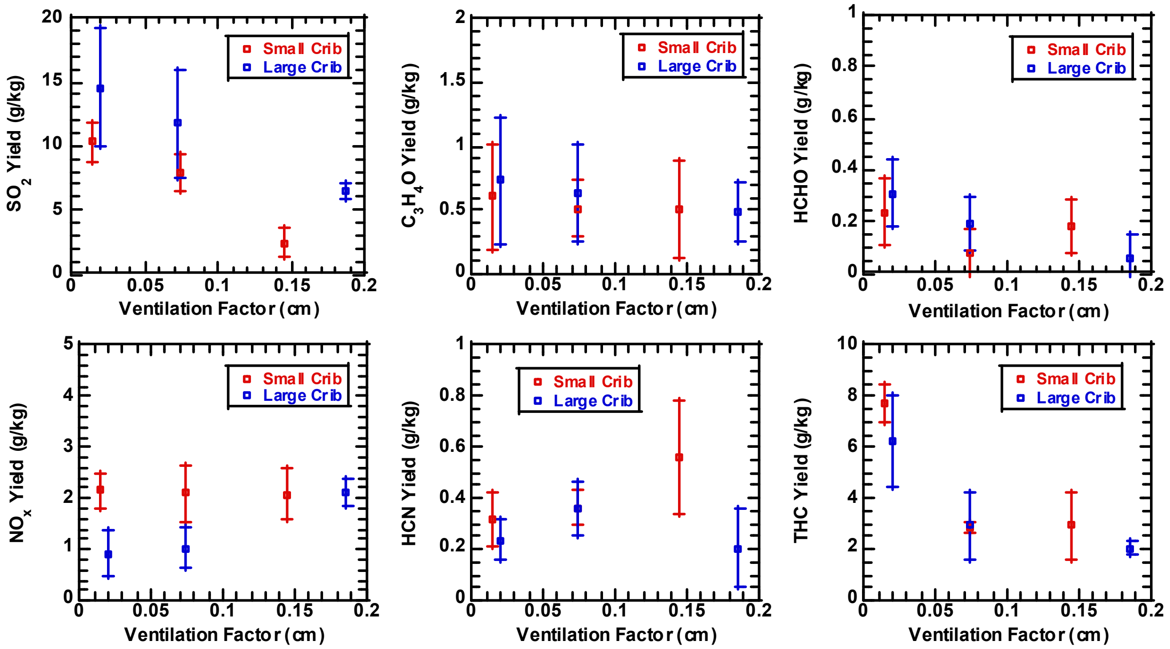

SI Figure 8: Trace gas yields against ventilation factor for large (blue) and small (red) cribs. Mean values and combined uncertainty with a coverage factor of two for repeated experiments reported.

Reference

(1) Bryant, R. A.; Bundy, M. F. The NIST 20 MW Calorimetry Measurement System for Large-Fire Research. *NIST* **2019**, *Technical Note 2077*. https://doi.org/10.6028/NIST.TN.2077.

(2) Burling, I. R.; Yokelson, R. J.; Griffith, D. W. T.; Johnson, T. J.; Veres, P.; Roberts, J. M.; Warneke, C.; Urbanski, S. P.; Reardon, J.; Weise, D. R.; Hao, W. M.; De Gouw, J. Laboratory Measurements of Trace Gas Emissions from Biomass Burning of Fuel Types from the Southeastern and Southwestern United States. *Atmos. Chem. Phys.* **2010**, *10* (22), 11115–11130. https://doi.org/10.5194/acp-10-11115-2010.

(3) Akagi, S. K.; Yokelson, R. J.; Wiedinmyer, C.; Alvarado, M. J.; Reid, J. S.; Karl, T.; Crounse, J. D.; Wennberg, P. O. Emission Factors for Open and Domestic Biomass Burning for Use in Atmospheric Models. *Atmospheric Chemistry and Physics* **2011**, *11* (9), 4039–4072. https://doi.org/10.5194/acp-11-4039-2011.

(4) Urbanski, S. P. Combustion Efficiency and Emission Factors for Wildfire-Season Fires in Mixed Conifer Forests of the Northern Rocky Mountains, US. *Atmospheric Chemistry and Physics* **2013**, *13* (14), 7241–7262. https://doi.org/10.5194/acp-13-7241-2013.

(5) Guérette, E.-A.; Paton-Walsh, C.; Desservettaz, M.; Smith, T. E. L.; Volkova, L.; Weston, C. J.; Meyer, C. P. Emissions of Trace Gases from Australian Temperate Forest Fires: Emission Factors and Dependence on Modified Combustion Efficiency. *Atmospheric Chemistry and Physics* **2018**, *18* (5), 3717–3735. https://doi.org/10.5194/acp-18-3717-2018.
